# Use of recombinant proteins for the diagnosis and prevention of *Mycoplasma bovis*: a systematic review

**DOI:** 10.3389/fvets.2024.1397145

**Published:** 2024-09-13

**Authors:** Camila Pachêco Gomes, Lucas Santana Coelho da Silva, Manoel Neres Santos Júnior, Maysa Santos Barbosa, Wanderson Souza Neves, Viviane Gomes Ribeiro, Bruno Lopes Bastos, Lucas Miranda Marques

**Affiliations:** ^1^Department of Microbiology, State University of Santa Cruz (UESC), Ilhéus, Brazil; ^2^Department of Biointeraction, Multidisciplinary Institute of Health, Federal University of Bahia, Salvador, Brazil; ^3^Department of Microbiology, Institute of Biomedical Sciences, University of São Paulo, São Paulo, Brazil

**Keywords:** systematic review, *Mycoplasma bovis*, diagnosis, vaccine, recombinant proteins

## Abstract

**Introduction:**

*Mycoplasma bovis* is a highly contagious pathogen that causes various diseases in herd animals, negatively impacting reproduction, production, and milk yield. Effective diagnostic methods and vaccine development are critical for controlling *M. bovis* outbreaks. This systematic review aimed to evaluate diagnostic alternatives and vaccine compounds based on recombinant proteins.

**Methods:**

Following the PRISMA protocol, a systematic search was conducted in the SciELO, PubMed, and CAPES Periodicals Portal databases. Inclusion criteria included studies published between 2008 and 2023 that involved (1) the use of recombinant proteins for *M. bovis* identification or vaccine production, (2) biological samples, (3) availability in the selected databases, (4) *in vitro* or *in vivo* experimental designs, and (5) English-language publications.

**Results:**

Ten of the initial 53 studies screened met the inclusion criteria. Of these, four studies focused on diagnostic approaches and six on vaccine development. Diagnostic studies predominantly used an indirect enzyme-linked immunosorbent assay (ELISA) with recombinant proteins, achieving over 90% sensitivity and specificity in detecting *M. bovis* infections. In contrast, the development of recombinant vaccines has shown limited success, with challenges in identifying effective adjuvants and optimizing conditions for protective immunity.

**Discussion:**

While recombinant protein-based diagnostics have proven effective, developing a successful vaccine against *M. bovis* remains elusive. Further research is necessary to refine vaccine formulations, including selecting suitable adjuvants and challenge models to enhance protective efficacy against *M. bovis* infections.

## 1 Introduction

*Mycoplasma bovis* is an important pathogen in beef and dairy cattle but is often overlooked. It primarily causes respiratory diseases such as mastitis, arthritis, keratoconjunctivitis, and otitis (1). It belongs to the *Mollicutes* class, a group of simple self-replicating bacteria characterized by minimal genomes (580–2,200 kb) and the absence of a cell wall (2). Once it infects an animal, it can persist within the herd for extended periods, possibly transmitting the pathogen to other animals for several weeks to months (3).

Diagnostic methods for *M. bovis*, including culture (4), PCR (5), and antigen-capture enzyme-linked immunosorbent assays (ELISAs) (6), only partially fulfill the need for a rapid diagnostic test to detect infection. Hence, sensitive and specific serological assays based on species-specific immunologically reactive antigens are needed (7).

*Mycoplasma bovis* is naturally resistant to antimicrobial agents targeting the cell wall and less susceptible to many commercially available antimicrobials, with resistant strains emerging worldwide (8–13). Therefore, without an effective vaccine or antimicrobial therapy, an alternative strategy for controlling *M. bovis* infection in cattle must separate infected and non-infected animals (14).

An alternative approach, in the form of recombinant antigens, may provide a successful avenue for both diagnosis and vaccination. Conserved recombinant proteins selected using *in silico* approaches might be more efficient than currently existing vaccines and diagnostic tests (15, 16). Thus, in this review, we aimed to investigate the use of recombinant proteins in the diagnosis of and vaccination against *M. bovis*.

## 2 Methods

### 2.1 Type of research

This systematic review was conducted by the Preferred Reporting Items for Systematic Reviews and Meta-Analyses (PRISMA) guidelines (17).

### 2.2 Research strategy

Searches were conducted in the Scientific Electronic Library Online (SciELO), PubMed, and CAPES Periodicals Portal databases to identify studies published between 2008 and 2023 that evaluated the use of recombinant proteins in the diagnosis of and vaccines against *Mycoplasma bovis*. Articles published and registered in these databases were identified using the Boolean operator related to the diagnosis [(recombinant protein* OR chimeric recombinant protein OR biosynthetic proteins) AND (diagnosis cattle mycoplasmas* OR diagnosis cattle mycoplasmosis OR diagnostic cattle mycoplasmas* OR diagnostic cattle mycoplasmosis)], and related to the vaccine [(recombinant protein* OR chimeric recombinant protein OR biosynthetic proteins) AND (vaccine cattle mycoplasmas* OR vaccine cattle mycoplasmosis OR potency of vaccine cattle mycoplasmas* OR potency of vaccine cattle mycoplasmosis)].

### 2.3 Eligibility criteria

A study was considered eligible for this systematic review if it met the following criteria: (1) used recombinant proteins in the diagnosis and vaccines against *Mycoplasma bovis*, (2) included biological samples, (3) were available in at least one of the selected databases, (4) were *in vitro* or *in vivo* studies related to the topic, (5) were published between 2008 and 2023, and (6) were available in English.

### 2.4 Study selection

CG, LS, MJ, MB, WN, VR, BB, LM

Five reviewers (LS, MJ, MB, WN, and VR) independently examined the titles and abstracts of articles to identify potentially relevant articles using the Rayyan QCRI platform^1^. The eligibility of the studies that met the inclusion criteria in this phase was confirmed by reading the full articles. Disagreements among the reviewers were resolved by a sixth reviewer (CG). Data extraction was performed by two independent reviewers (CG and LS) using a standardized form developed by the authors based on the PRISMA checklist of the University of Oxford (17). The extracted data included information related to the publication, study design, location, and year of publication.

## 3 Results

### 3.1 Selected studies

**Figure 1:**
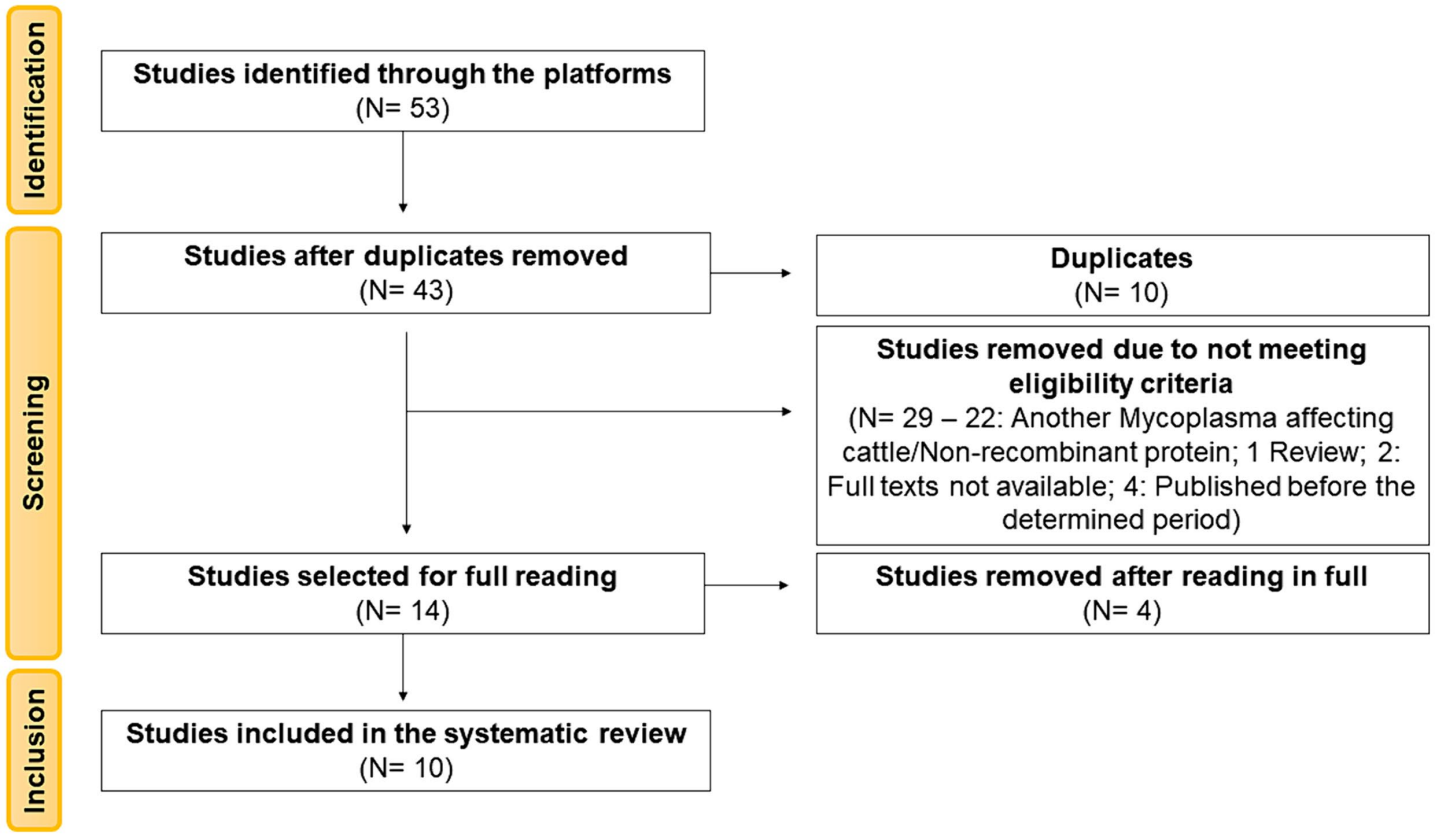
PRISMA 2020 flowchart showing the article selection process used for the systematic review.

A total of 53 articles were identified from the searched databases. Ten articles (18.87%) were removed as duplicates during the screening stage. Of the remaining 43 articles (81.13%), based on their titles and abstracts, 29 articles still needed to meet the previously defined eligibility criteria. Among these, 22 articles were related to another Mycoplasma affecting cattle or did not involve recombinant proteins, 1 article did not include biological samples as it was a review article, two articles had full texts that were not available in the databases, and four articles were published before the determined period, and therefore, were not considered for full reading. The remaining 14 articles were read in total, of which four were excluded for not aligning with the objective of this review. Therefore, this review was based on the analysis of 10 articles (Figure 1).

### 3.2 Characteristics of the studies

**Figure 2:**
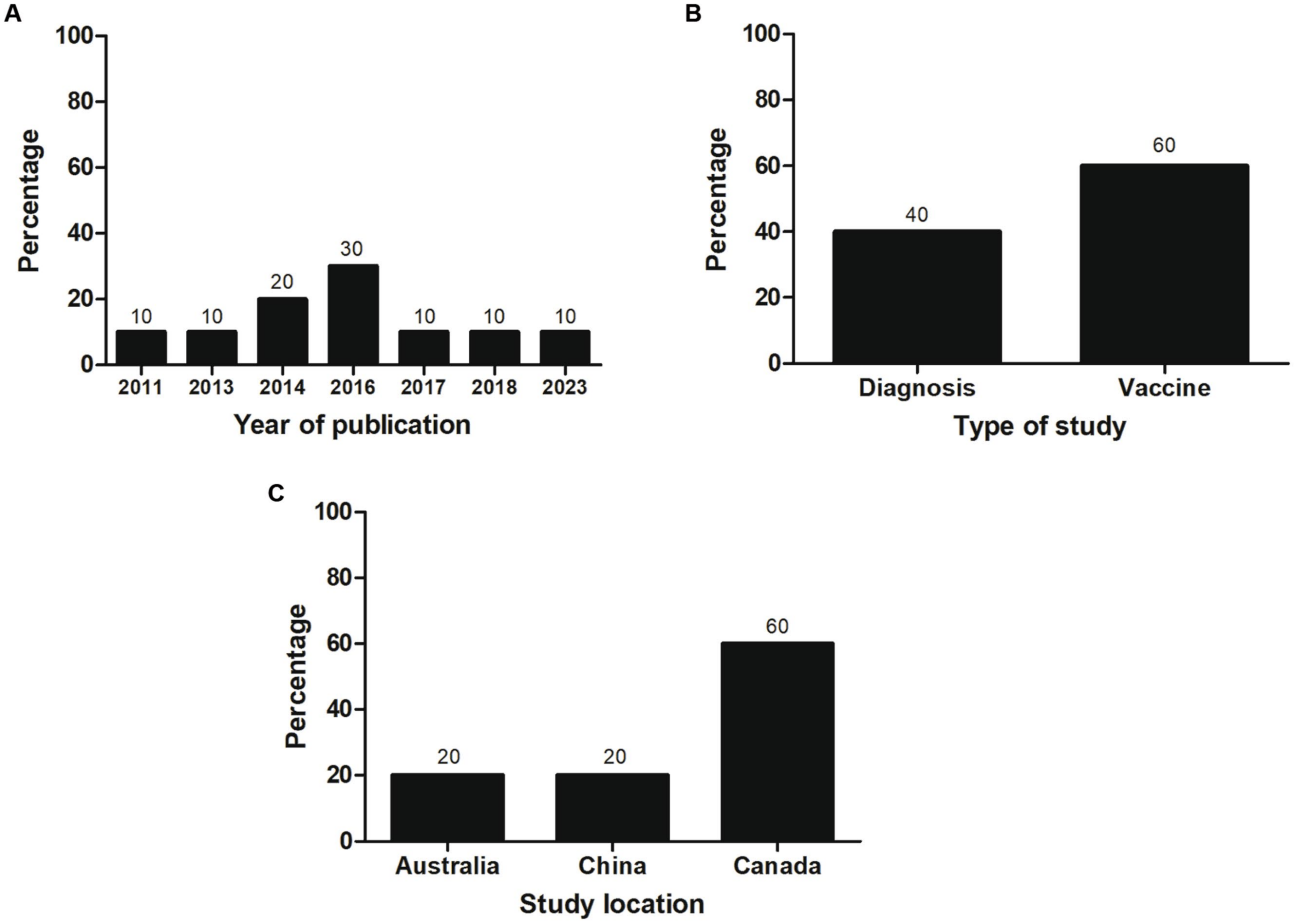
General characteristics of the studies included in the systematic review (*n* = 10). The percentage of studies by **(A)** year of publication, **(B)** type of study, and **(C)** study location is shown.

Most of the selected studies (*n* = 6; 60%) focused on vaccines, followed by studies related to diagnosis (*n* = 4; 40%). Most studies (*n* = 3; 30%) were published in 2016. Most investigations were conducted in Canada (60%) and corresponded to vaccine articles, all carried out by the same research group. An equal number of investigations (*n* = 2; 20%) related to diagnosis were performed at Chinese and Australian institutes (Figure 2). Individual data were categorized and extracted based on references (Tables 1, 2). Significant variability existed among the recombinant antigens used for diagnosis and vaccines against *M. bovis*.

**Table 1:** Summary of studies related to the diagnosis included in the systematic review (*n* = 4).

Reference	Country of study	Year	Type of test	Sample	Recombinant proteins	Sensitivity	Specificity
(7)	Australia	2014	Indirect IgM and IgG ELISA	Serum	Micoplasma immunogenic lipase A (MilA)	IgM 97.1% IgG 92.86%	IgM 63.3% IgG 98.7%
(20)	Australia	2016	Indirect IgG ELISA	Serum	Micoplasma immunogenic lipase A (MilA)	94.3% (CI 95%, 89.9% a 99.6%)	94.4% (CI 95%, 90.3% a 99.6)
(46)	China	2014	Indirect ELISA and Western blot	Serum	rPDHB	–	rPDHB-based iELISA did not react with other pathogens evaluated, except *M. agalactie*
(2)	China	2016	Indirect ELISA	Serum	rMbovP116, rDLD, rTkt, rpepP, rMbovP275, rMbovP579, rMbovP739, rpepA	90.2% (CI 95%, 83.7%, 94.3%)	97.8% (CI 95%, 88.7%, 99.6%)

CI, confidence interval.

**Table 2:** Summary of vaccine-related studies included in the systematic review (*n* = 6).

Reference	Country of study	Year	Type of vaccine	Recombinant antigen	Adjuvant used	Type of test	Vaccination route/No of doses/time interval	Age of vaccinated animals	Efficacy
(34)	Canada	2011	Subunit-based protective	GAPDH-BMAP28 (sGap-M), GAPDH-indolicidina (sGap-I) e GAPDH-TAP (Gap-T)	Host defense peptides (HDP): BMAP28, indolicidina, TAP	*in vitro*	–	–	Yes
(16)	Canada	2013	Subunit-based protective	GAPDH-indolicidina (sGap-I)	30% Emulsigen^™^	*in vivo*	Subcutaneous or intradermal/two/42 days	6 to 8 months	No
(33)	Canada	2016	Subunit-based protective	PdhA, PepA, Tuf, P48, P81, OppA, LppB, PepQ, O256, DeoB	TriAdj (Polyphosphazene EP3 + poly I:C + IDR peptide 1,002) Emulsigen^™^ + poly I:C + IDR peptide 1,002	*in vivo*	Subcutaneous or Intradermal/two/21 days	6 to 8 months	Partial
(30)	Canada	2017	Subunit-based protective	PdhA, PepA, Tuf, P48, P81, OppA, LppB, PepQ, O256, DeoB	Emulsigen^™^ + poly I:C + IDR peptide 1,002	*in vivo*	Subcutaneous/two/40 days	6 to 8 months	No
(31)	Canada	2018	Subunit-based protective	PdhA, Tuf, PepA, LppB, O256, OppA, DeoB, P81, and PepQ	Montanide ISA61^™^ VG e curdlan	*in vivo*	Subcutaneous/two/21 days	6 to 8 months	No
(32)	Canada	2023	Subunit-based protective	Hyp-1, ISMbov1, LplC, DppB, Mem, L21, Nfo, Hyp-2, MgtA, PAR, Hyp-3, Hyp-4, Hyp-5, FbpB, SpII, Gmk, L7-L12, UgpE, Zip	60% Montanide ISA65 VG-Curdlan	*in vivo*	Subcutaneous/two/21 days	6 to 8 months	No

## 4 Discussion

Cattle farming is fundamentally essential for the agricultural sector, and the rapid identification and prevention of diseases are becoming increasingly important due to the significant economic consequences of animal disease outbreaks. Besides the impacts on national and international trade, diseases are also responsible for direct losses to producers due to reduced production, increased mortality, or treatment costs (18).

Under natural conditions, *M. bovis* infection is challenging to identify and is easily mistaken for contagious pleuropneumonia because of similar clinical symptoms and pathological changes. Therefore, laboratory differential diagnosis is the best method for identifying *M. bovis* infections. Generally, serological diagnosis is more sensitive than *M. bovis* isolation, especially in chronic cases or animals treated with antibiotics (1). Currently, some commercial indirect ELISA kits have been used for this purpose; however, most kits are based on whole-cell proteins, and the effects on detecting *M. bovis* infection in different geographical regions have not yet been verified, thus requiring complementary techniques for accurate diagnosis (19). Therefore, using highly pure specific antigens with high antibody affinities, such as recombinant antigens, may increase diagnostic accuracy. A simple, rapid, specific, and sensitive diagnostic technique is preferred for the intensive surveillance of infectious animal diseases, such as *M. bovis*.

We observed a predominance of studies that developed ELISAs for *M. bovis* detection and achieved higher success using recombinant proteins than commercial ELISA kits. Wawegama et al. (7) identified an ideal antigen for *M. bovis* detection with no antigenic variation. They sought to identify an immunogenic protein by western blotting using sera from *M. bovis*-specific cattle, characterize its function, develop an indirect ELISA (iELISA) using this protein, and evaluated its sensitivity using a panel of sera collected from experimentally infected calves. A novel 226 kDa protein, Mycoplasma immunogenic lipase A (MilA), was identified and demonstrated immunoreactive with *M. bovis*-specific calf sera. The IgG ELISA based on a MilA fragment had a sensitivity of 92.86% and specificity of 98.7%. It demonstrated that it was a rapid and convenient method with reproducibility for detecting *M. bovis*-infected cattle under experimental conditions. The authors continued their study, and in 2016 (20), they assessed the performance of IgG ELISA using the MilA antibody with a diagnostic assay in experimentally infected young calves, beef cattle, and confined cattle. They evaluated its potential as a serological assay for detecting prior *M. bovis* infections under field conditions. IgG ELISA using MilA could detect seroconversion after *M. bovis* infection in calves and prior infection in adult cattle under field conditions, estimating the diagnostic test performance (sensitivity and specificity). This assay’s high sensitivity and specificity under field conditions suggest its potential usefulness for screening herds to assist in developing enhanced biosecurity measures for *M. bovis* in beef herds. It also serves as a tool for epidemiological investigations to estimate the impact of *M. bovis* on animal welfare and productivity better and to identify risk factors for infection and disease, as well as priority areas for better understanding and control of this pathogen.

Proteome analysis is a proper complementary method for studying pathogens as it aids in genome annotation and protein identification (21). Immunoproteomics, which combines conventional proteomics with serology, is a powerful method for identifying immunodominant antigens with diagnostic and protective value (22). Sun et al. (23) used proteomics to screen the immunogenic proteins of an *M. bovis* strain isolated in China. After analyzing the immunogenicity and antigenicity of the 19 identified proteins, one protein was selected. A serum antibody detection iELISA was established based on the E1 beta subunit of a recombinant antigenic protein expressed prokaryotically in the pyruvate dehydrogenase complex (rPDHB). This study confirmed the viability of rPDHB as a diagnostic antigen and demonstrated its high sensitivity and specificity. This study marked the identification of *M. bovis* PDHB, which displayed excellent immunogenicity and repeatability. It offered a supplementary diagnostic tool for infectious bovine pneumonia resulting from *M. bovis*, contributing to the progression of epidemiological research and the implementation of relevant quarantine measures.

Considerable efforts have been made to elucidate the antigenic structures of *M. bovis*, and some relevant recombinant proteins have been identified, as demonstrated in this review. However, studies on existing proteins and their recombinants, as well as on the use of immunoproteomics, need to be more comprehensive to identify potential antigens containing multiple T and B cell epitopes. Therefore, combining immunoproteomics, immunoinformatics, conventional gene expression, and subsequent immunological confirmation provides an effective method for comprehensively characterizing antigenic proteins (24).

In 2016, Khan and colleagues (2) assembled a global antigenic profile for *M. bovis* using immunoproteomics and immunoinformatics. They aimed to identify promising candidate proteins in *M. bovis* using gene expression analyses and other serological methods. Among the 16 studied proteins, they observed that of the eight identified *M. bovis* antigens expressed in *E. coli*, MbovP579 was confirmed to be a conserved, sensitive, and specific antigen present in field isolates as well as the attenuated vaccine strain *M. bovis*-150. Consequently, an iELISA based on the recombinant protein MbovP579 (rMbovP579) could effectively detect specific *M. bovis* IgG in animals with acute or chronic infections, likely aiding in the early detection of *M. bovis* infection.

After observing the results and conclusions obtained from the four articles (2, 7, 20, 23) regarding the diagnosis and use of recombinant proteins, we identified that the lack of knowledge about the antigenic properties of *M. bovis* proteins hinders further studies and the effective control of bovine infections using immunological approaches. Thus, the screening, identification, and use of *M. bovis* proteins with excellent immunogenicity are necessary to renew, update, and improve diagnostic techniques to obtain a highly sensitive and specific method for the early diagnosis of *M. bovis* infection, as presented here.

Beyond effective and early diagnosis, prevention through vaccination has become a necessary intervention to study, as vaccines represent an intervention strategy with the best cost–benefit ratio applied in public health. Biotechnological advances in various fields have contributed to developing safer and more effective formulations. In addition, the application of biotechnological tools in vaccine development has changed how we think about and produce these agents for use in humans and animals (25).

Various attempts to control *M. bovis* infections through vaccination have resulted in mixed outcomes. Numerous challenges have hindered the success of vaccines in preventing *M. bovis* infections. Among these challenges are the colonization of the upper respiratory tract in young animals, the quality and modulation of the host immune response, the role of other respiratory pathogens, and the need for a challenge model reproducing the disease observed in the field (15). Using conserved recombinant proteins selected using *in silico* approaches as vaccine components might be a better choice because of the limited protection offered and adverse reactions caused by traditional methodologies.

In the absence of mycoplasma research, many efforts have been made to produce potential recombinant proteins for the prevention and/or detection of animal mycoplasmosis using immunoinformatics for antigen selection. The construction of vectors encoding chimeric proteins constitutes a promising area for immunodiagnostics in mycoplasma studies and vaccines and therapeutic bioproducts in other fields (26–29).

A group of researchers from Canada sought, through the analysis of various proteins and methodologies, to identify protective antigens, effective adjuvants, and information regarding the quality of immune responses necessary for protection (16, 30–34). The possibility of using proteins such as glyceraldehyde-3-phosphate dehydrogenase (GAPDH) as vaccine targets has been described for several microorganisms, including *Schistosoma mansoni*, *Streptococcus dysgalactiae*, *Candida albicans*, *Mycoplasma bovis*, and *Brucella abortus* (35–39), and GAPDH appears to be a protective antigen. In addition to its well-characterized role in glycolysis, GAPDH has been implicated in the virulence of pathogenic microorganisms, and owing to its location on the cell surface, it acts as an adhesin for the colonization of tissue surfaces in both pathogenic and non-pathogenic normal microbiota. These novel properties of GAPDH make it a target for pathogenic studies and a candidate for developing vaccines against various diseases. In 2011, Van der Merwe and collaborators (34) reported the isolation and use of GAPDH protein from *M. bovis* as a test antigen to develop a vaccine to protect confined animals from *M. bovis* and related diseases. Several new immunological modulators have been tested in vaccine studies, some of which are host defense peptides (HDPs). HDPs are small proteins in the host’s innate immune system, possess antimicrobial activities, and can act as adjuvants. These new compounds have been used as chimeric proteins composed of viral antigens fused to HDPs to promote immune responses. The first step in using the *M. bovis* GAPDH protein and HDP as components of a vaccine was to construct the chimeric proteins *M. bovis* GAPDH-HDP: GAPDH-BMAP28 (sGap-M), GAPDH-indolicidin (sGap-I), and GAPDH-TAP (Gap-T), which retained the properties associated with the individual components, that is, GAPDH enzymatic antimicrobial activities and HDP properties. Vaccination with a chimeric protein composed of *M. bovis* GAPDH and HDP as an adjuvant could potentially result in increased immune responses to the GAPDH fraction and thus offer protection against *M. bovis* infection.

In 2013, the same research group (16) conducted a study in which they reported using Gap-I, a chimeric protein composed of *M. bovis* GAPDH and the host defense peptide indolicidin, which maintained the properties of the individual components. Thus, they used vaccines consisting of a plasmid encoding the Gap-I protein formulated in PBS, the Gap-I protein, and *M. bovis* cell extracts formulated with synthetic oligonucleotides (CpG2007) to investigate whether these vaccines could protect confined cattle from an experimental challenge. Vaccination with Gap-I in protein and DNA or mixed with *M. bovis* extract failed to safeguard the confined cattle. These results suggest that GapI vaccination may increase lung pathology. Despite the solid humoral immune responses obtained, searching for suitable protective antigens continues, and new approaches are needed.

Therefore, identifying suitable antigenic and immunogenic components is essential for vaccine success. Despite these studies, researchers still need more information on the antigens and adjuvants necessary to confer animal protection. Based on the previously obtained results, a different adjuvant might increase vaccine effectiveness, or not all the antigens needed for protection were present in the experimental vaccines (40). Prysliak and Perez-Casal (33) employed a proteomic approach to develop a subunit-based protective vaccine. This approach resulted in the identification of several proteins, namely, four surface-exposed proteins (P48, P81, OppA, and LppB), three proteases (PepA, PepQ, and O256), two proteins involved in sugar metabolism (PdhA and DeoB), and one protein involved in protein synthesis (Tuf). These proteins were first tested against serum samples from animals challenged with *M. bovis*, and the results indicated that there were significant humoral responses for all ten proteins in post-challenge serum samples compared to those in pre-challenged animal sera. Subsequently, TriAdj, a new experimental vaccine adjuvant with three components developed at the VIDO-InterVac, was used to formulate these candidates to evaluate humoral and cell-mediated immune responses in vaccinated animals. Furthermore, they compared the immune responses following formulation with TriAdj with responses measured in animals immunized with a mixture of a commercial adjuvant (Emulsigen^™^) and two of the TriAdj components, namely, polyinosinic: polycytidylic acid (poly I: C) and innate defense regulator cationic peptide (IDR) 1,002 (VQRWLIVWRIRK). In this last trial, they detected significant IgG1 humoral immune responses to 8 out of 10 *M. bovis* proteins and IgG2 responses to 7 out of 10 proteins. Thus, they concluded that the commercial adjuvant formulated with poly I: C and the IDR 1002 peptide is the best formulation for the experimental vaccine, becoming an improved version of previous studies, which adds new antigens and a new adjuvant formulation that may result in adequate protection. However, when testing this formulation and challenging the animals using a coinfection model with bovine herpesvirus 1 (BHV-1)/*M. bovis*, although PBMC proliferation and cytokine responses to vaccine antigens were insignificant, humoral responses revealed that eight antigens elicited a balanced IgG1/IgG2 response. This implies that these proteins may play an essential role in protecting against *M. bovis* by opsonizing the bacterium and promoting its phagocytosis during infection. However, this did not confer protection against *M. bovis* during the experimental challenge (30).

Following these studies, Prysliak et al. (31) sought to identify *M. bovis* antigens that could trigger a Th-17 protective response. They tested a vaccine containing *M. bovis* proteins formulated with Montanide ISA61^™^ VG and curdlan, an inducer of Th-17 responses. After vaccination, the animals were challenged with a BHV-1/*M. bovis* co-infection model. They detected IL-17 and other cytokines in PBMC supernatants. Additionally, they observed proliferative antibodies and PBMC responses to the antigens. Despite significant humoral and cellular responses to the antigens, detection of Th-17 immune responses, and slight reductions in the proportion of lung lesions and weight loss in the vaccinated group, the authors concluded that Th-17 responses to the antigens used here were not protective. Compared with previously obtained results (30), there were no significant changes in serum cytokine levels after the challenge. This was not entirely surprising, given the low degree of disease observed after the challenge. This suggests new approaches are necessary to identify protective antigens and effective adjuvants.

The identification of new antigens for use in vaccines was then accelerated by employing the reverse vaccinology approach (41), an unbiased methodology wherein various bioinformatic approaches are used to analyze all potential candidate proteins encoded by a bacterium. In another attempt, Prysliak et al. (32) explored the role of complement-fixing antibodies in the death of *M. bovis in vitro* and whether animals vaccinated with proteins that induce complement-fixing antibodies would be protected against an experimental challenge using various antigens of *M. bovis* identified through reverse vaccinology approaches. They found antibodies against some of these proteins fixed the complement system and killed *M. bovis in vitro*. When testing the role of these antibodies in protecting vaccinated cattle against an experimental challenge, the results indicated a lack of protection. However, the antigens might not have triggered a protective response, and the antibody levels at the site of infection might have to be considered.

To enhance the immune responses in the lungs of animals, an alternative could be using the intranasal route instead of subcutaneous or intradermal administration of the vaccine. In a study conducted by Zhang et al. (42), an intranasally administered vaccine composed of attenuated strains of *M. bovis* provided protection. Thus, these results suggest that recombinant antigens administered intranasally may reach antigen-presenting cells in the lungs, resulting in a more effective local immune response. Therefore, the next steps in this study would involve delivering the antigens intranasally and measuring the immune response in nasal fluid, followed by a challenge.

Since the first attempt to develop vaccines against calf pneumonia was reported (43), various formulations and attempts using different methodologies have been tested, resulting in mixed outcomes. Still, they have yet to be entirely adequate so far. However, researchers are increasingly seeking more modern approaches, such as those described in this study, which have shown some beneficial results, such as weight gain, slightly fewer lung lesions, and a strong humoral immune response (16, 31, 40). Despite the observed helpful results, it was insufficient to confer protection against the *M. bovis* challenge. One of the reasons for the inadequate challenge might be a much lower infectious dose compared to those used in other *M. bovis* vaccine studies (40, 44, 45). Thus, attempts with different recombinant antigens, different adjuvants, co-infection and challenge models, and routes of administration have been tested, and researchers have already planned a new route of administration to be tested. However, in addition to reviewing and evaluating all these aspects, it is also essential to consider the animals’ age, as all studies presented here used animals in the same age range (6 to 8 months). The ideal age to vaccinate cattle is crucial for *M. bovis* vaccines and is often a barrier to adequate protection against infections with this pathogen.

The articles included in this review then present the most up-to-date data on the use of recombinant proteins in diagnosing and developing vaccines against *M. bovis*, significantly contributing to improving knowledge in this field.

## 5 Conclusion and future perspectives

The present study systematically reviewed approaches used regarding recombinant proteins for diagnosing and developing protective vaccines against *M. bovis*. A practical and rapid diagnostic method is necessary to identify animals infected with *M. bovis* and minimize livestock losses. Here, we observed a predominance of successful studies using modern approaches to identify recombinant proteins obtained from a panel of sera and isolated strains of *M. bovis*, which were used to develop indirect ELISAs to detect *M. bovis*. This serological technique is currently the most widely used to detect specific antibodies and has advantages in using many samples for screening, making it ideal for surveillance and biosecurity programs. On the other hand, despite numerous efforts, developing protective recombinant vaccines to control *M. bovis* infection has not been successful. Much research in this area is still needed, especially regarding the development of an animal challenge model, the identification of antigens that could trigger antibodies to protect animals from the challenge, the best route for immunization, and the ideal age for vaccinating animals, which remains a barrier to adequate protection. Thus, a significant challenge researchers face in developing new immunization strategies is designing vaccines that induce an appropriate immune response to confer immunity, especially against intracellular pathogens, particularly those that establish chronic infections, often throughout life. To achieve this, it is necessary to understand the biology of highly conserved antigens involved in pathogenesis and the immunological mechanisms that need to be triggered for protection to rationally design vaccine strategies that can overcome the low protective immunity naturally generated by infection. Hopefully, the use of reverse vaccinology and bioinformatics tools will enable the proteomic analysis of *M. bovis* and, consequently, the detection of new proteins that can be used not only as diagnostic biomarkers but also in the development of a potent vaccine for the effective control of *M. bovis* infections.

## Data Availability

The raw data supporting the conclusions of this article will be made available by the authors, without undue reservation.

## Footnotes

^1^ http://rayyan.qcri.org/
